# Supplementary material for: Skill deficits among foreign-educated immigrants: Evidence from the U.S. PIAAC
Source: PLoS One. 2022 Aug 30;17(8):e0273910. doi: 10.1371/journal.pone.0273910 (PMC9426902; doi:10.1371/journal.pone.0273910)
Supplement: S1 Table — (a) Standardized differences in literacy or numeracy test scores between foreign- and U.S.-educated immigrants, with and without an adjustment for years of U.S. residence. Negative numbers imply a U.S.-educated advantage. (b) Foreign-educated odds ratios of scoring at a higher level on the four-level PST test relative to U.S.-educated immigrants, with and without an adjustment for years of U.S. residence. Odds ratios less than one imply a U.S.-educated advantage. Residence is a categorical variable with ten different values. All comparisons are adjusted for age and self-assessed English reading ability. Standard errors are in parentheses. * p < 0.05, ** p < 0.01. (DOCX) [file pone.0273910.s001.docx]

**Table S1. Effect of years of residence on test-score differentials between foreign- and U.S-educated immigrants.**

|  | I | II |
| --- | --- | --- |
|  | Full Sample, No Education Controls | Full Sample, Education Controls |
| 1. Literacy |  |  |
| without residence control | -0.33** | -0.22** |
|  | (0.08) | (0.07) |
| with residence control | -0.46** | -0.20* |
|  | (0.08) | (0.08) |
|  |  |  |
| 1. Numeracy |  |  |
| without residence control | -0.23** | -0.10 |
|  | (0.07) | (0.06) |
| with residence control | -0.42** | -0.12 |
|  | (0.08) | (0.08) |
|  |  |  |
| 1. PST |  |  |
| without residence control | 0.46** | 0.50** |
|  | (0.08) | (0.09) |
| with residence control | 0.34** | 0.56** |
|  | (0.07) | (0.13) |

(a) Standardized differences in literacy or numeracy test scores between foreign- and U.S.-educated immigrants, with and without an adjustment for years of U.S. residence. Negative numbers imply a U.S.-educated advantage. (b) Foreign-educated odds ratios of scoring at a higher level on the four-level PST test relative to U.S.-educated immigrants, with and without an adjustment for years of U.S. residence. Odds ratios less than one imply a U.S.-educated advantage. Residence is a categorical variable with ten different values. All comparisons are adjusted for age and self-assessed English reading ability. Standard errors are in parentheses. * *p* < 0.05, ** *p* < 0.01
