# Supplementary material for: Skill deficits among foreign-educated immigrants: Evidence from the U.S. PIAAC
Source: PLoS One. 2022 Aug 30;17(8):e0273910. doi: 10.1371/journal.pone.0273910 (PMC9426902; doi:10.1371/journal.pone.0273910)
Supplement: S2 Table — The first two columns display the coefficients from an OLS regression of the log of hourly wages on different sets of worker characteristics. The differences between the foreign-educated coefficients in the first and second columns are displayed in the bottom section. The last two columns display the odds ratios from ordered logit regressions of occupational skill requirements on different sets of worker characteristics. The ratios of the foreign-educated coefficients in the third and fourth columns are displayed in the bottom section. Standard errors are in parentheses. * p < 0.05, ** p < 0.01. To comply with government disclosure restrictions, sample sizes have been rounded to the nearest 10. (DOCX) [file pone.0273910.s002.docx]

**Table S2. Effect of controlling for test scores on the immigrant-native wage and skilled employment gaps, with immigrants separated by years of U.S. residency.**

|  | DV = Wages | | DV = Occupation Skill Level | |
| --- | --- | --- | --- | --- |
|  |  |  |  |  |
|  | Baseline | Plus Test Scores | Baseline | Plus Test Scores |
| Immigrants (ref. = Natives) |  |  |  |  |
| U.S.-educated |  |  |  |  |
| Residence <15 years | 0.05 | 0.06 | 1.41 | 1.69 |
|  | (0.07) | (0.07) | (0.39) | (0.53) |
| Residence 15+ years | 0.04 | 0.07 | 1.46 | 1.70* |
|  | (0.04) | (0.04) | (0.28) | (0.32) |
| Foreign-educated |  |  |  |  |
| Residence <15 years | -0.14** | -0.09 | 0.59* | 0.75 |
|  | (0.05) | (0.05) | (0.16) | (0.21) |
| Residence 15+ years | -0.10 | -0.02 | 0.82 | 1.03 |
|  | (0.06) | (0.06) | (0.21) | (0.25) |
| Age (ref. = 18-24) |  |  |  |  |
| 25-34 | 0.29** | 0.27** | 1.64* | 1.66* |
|  | (0.02) | (0.02) | (0.27) | (0.29) |
| 35-44 | 0.49** | 0.49** | 1.85** | 2.00** |
|  | (0.03) | (0.03) | (0.30) | (0.34) |
| 45-54 | 0.51** | 0.53** | 1.64* | 1.95** |
|  | (0.03) | (0.02) | (0.27) | (0.35) |
| 55-64 | 0.59** | 0.62** | 1.93** | 2.61** |
|  | (0.03) | (0.03) | (0.32) | (0.46) |
| Education (ref. = Less Than HS) |  |  |  |  |
| High School (HS) | 0.21** | 0.12** | 1.82** | 1.51* |
|  | (0.03) | (0.03) | (0.28) | (0.24) |
| Some College | 0.36** | 0.23** | 4.21** | 3.08** |
|  | (0.03) | (0.03) | (0.82) | (0.66) |
| Bachelor's | 0.66** | 0.44** | 18.03** | 11.64** |
|  | (0.04) | (0.04) | (3.16) | (2.38) |
| Advanced | 0.82** | 0.57** | 69.09** | 44.44** |
|  | (0.05) | (0.06) | (20.02) | (13.92) |
|  |  |  |  |  |
| Read English (ref. = Very Well) |  |  |  |  |
| Well | -0.03 | 0.02 | 0.62** | 0.77 |
|  | (0.03) | (0.03) | (0.10) | (0.13) |
| Not Well | -0.21** | -0.12* | 0.45** | 0.58* |
|  | (0.05) | (0.05) | (0.14) | (0.17) |
| Not at All | -0.33** | -0.22* | 0.18** | 0.20** |
|  | (0.08) | (0.09) | (0.07) | (0.08) |
| Test Scores |  |  |  |  |
| Literacy |  | -0.003 |  | 1.356* |
|  |  | (0.034) |  | (0.16) |
| Numeracy |  | 0.127** |  | 0.745** |
|  |  | (0.029) |  | (0.08) |
| PST (ref. = Level 0) |  |  |  |  |
| Level 1 |  | 0.04 |  | 1.93** |
|  |  | (0.03) |  | (0.31) |
| Level 2 |  | 0.09* |  | 2.74** |
|  |  | (0.04) |  | (0.63) |
| Level 3 |  | 0.13 |  | 3.91 |
|  |  | (0.07) |  | (1.56) |
| Constant | 2.19** | 1.68* |  |  |
|  | (0.04) | (0.09) |  |  |
|  |  |  |  |  |
| *r*^2^ | 0.35 | 0.39 |  |  |
| N | 4,160 | 4,160 | 4,160 | 4,160 |
|  |  |  |  |  |
| Δ Foreign-Educated: residence <15 |  | 0.05** |  | 1.29** |
|  |  | (0.02) |  | (0.07) |
|  |  |  |  |  |
| Δ Foreign-Educated: residence 15+ |  | 0.08** |  | 1.26** |
|  |  | (0.02) |  | (0.07) |

The first two columns display the coefficients from an OLS regression of the log of hourly wages on different sets of worker characteristics. The differences between the foreign-educated coefficients in the first and second columns are displayed in the bottom section. The last two columns display the odds ratios from ordered logit regressions of occupational skill requirements on different sets of worker characteristics. The ratios of the foreign-educated coefficients in the third and fourth columns are displayed in the bottom section. Standard errors are in parentheses. * *p* < 0.05, ** *p* < 0.01. To comply with government disclosure restrictions, sample sizes have been rounded to the nearest 10.
