# Supplementary material for: Skill deficits among foreign-educated immigrants: Evidence from the U.S. PIAAC
Source: PLoS One. 2022 Aug 30;17(8):e0273910. doi: 10.1371/journal.pone.0273910 (PMC9426902; doi:10.1371/journal.pone.0273910)
Supplement: Attachment — Submitted filename: Response to Reviewers.docx [file pone.0273910.s003.docx]

I thank both reviewers for their very helpful suggestions, all of which I have incorporated into the revised manuscript.

Response to Reviewer #1

The first reviewer’s point that the difference in foreign degrees could be due to different accreditation standards and practices across countries, and that such issues would perhaps decline in importance as immigrants acculturate, is well taken. I revised the manuscript to address this issue in three ways:

First, I added to the Discussion section the role of time in potentially reducing the legal and social obstacles to advancement for foreign-educated immigrants.

Second, I conducted a new regression analysis that splits the foreign-educated immigrants into two groups of roughly equal size – those who have been in the U.S. for less than 15 years, and those who have been in the U.S. for at least 15 years. This analysis shows that the wage and skilled-employment gaps that remain after controlling for PIAAC scores are substantial for recent arrivals but fade to approximately zero over time. This result is consistent with a model in which structural factors initially play some role in labor market disparities, but skill differences gradually become the dominant factor. I considered putting this new regression analysis in the main text, but ultimately I decided to include it as a supplemental table (Table S2) that I reference in the Discussion section. The reason is that the analysis requires splitting an already small sample of foreign-educated immigrants in half, and I want to be careful about not attempting to draw strong conclusions from regression results that have high standard errors.

Third, I re-emphasized in the concluding paragraph that further research with larger datasets can hopefully address the reviewer’s note that country- and region-based variation are important in establishing the value of foreign degrees.

Response to Reviewer #2

1) I have added text to the regression portion of the methods section that clarifies earnings are self-reported and converted by PIAAC into an hourly wage based on responses to other questions about work time. I have also added that earnings do not include self-employment.
2) I have added text to the same portion of the methods section indicating that there are no meaningful changes to coefficients when including very high and low earnings, but the model fit (as measured by r-squared) does go down.
3) Thanks for pointing this out. The sample size had been changing due primarily to some missing occupational skill scores. I have re-run the wage and employment regressions with a consistent sample and updated Tables 6 and 7 accordingly. The results are little changed, but I agree it is better to keep the sample sizes the same across models.
4) Good thought. I have added a paragraph to the Key Terms section acknowledging how common 1.5 gen immigrants are in the U.S.-educated group and citing researching indicating that they typically outperform the 1.0 generation. I explain that it’s not practical to separate 1.5 from 1.0 in this study; however, I have added a table in the Supplemental Information section showing that test scores differences between foreign- and U.S.-educated immigrants are not a function of different amounts of time spent in the U.S.
5) I have added or modified text in several places to be clearer about what I mean by “imprecision,” including at the end of the Introduction.
6) Thanks. I have incorporated Oreopoulos into the discussion, and in the same section I’ve added citations to a few other papers that discuss potential legal and social obstacles to immigrant advancement.
7) They’re standard measures of skill that have been used in wage regressions in the past. I’ve modified the text on that page to make this clearer, and I’ve rewritten the description of the cited paper to say explicitly that the PIAAC scores have been found to be significant determinants of earnings.
8) I rewrote the end of that paragraph to explain in more detail why sample size limitations preclude detailed analyses of subgroups.
9) I added and modified text in that paragraph to explain that skilled employment results are similar to the wage results in that they show test scores can explain much of the disparity, but additional factors cannot be ruled out.
